# RNA-sequencing analysis of umbilical cord plasma microRNAs from healthy newborns

**DOI:** 10.1371/journal.pone.0207952

**Published:** 2018-12-03

**Authors:** Gary P. Brennan, Dimitrios M. Vitsios, Sophie Casey, Ann-Marie Looney, Boubou Hallberg, David C. Henshall, Geraldine B. Boylan, Deirdre M. Murray, Catherine Mooney

**Affiliations:** 1 Department of Physiology & Medical Physics, Royal College of Surgeons in Ireland, Dublin, Ireland; 2 FutureNeuro Research Centre, Royal College of Surgeons in Ireland, Dublin, Ireland; 3 European Molecular Biology Laboratory–European Bioinformatics Institute, Wellcome Trust Genome Campus, Hinxton, Cambridge CB10 1SD, United Kingdom; 4 INFANT Research Centre, University College Cork, Cork, Ireland; 5 Department of Paediatrics & Child Health, University College Cork, Cork, Ireland; 6 Neonatology, Karolinska University Hospital, Stockholm, Sweden; 7 School of Computer Science, University College Dublin, Belfield, Dublin 4, Ireland; Universitat des Saarlandes, GERMANY

## Abstract

MicroRNAs are a class of small non-coding RNA that regulate gene expression at a post-transcriptional level. MicroRNAs have been identified in various body fluids under normal conditions and their stability as well as their dysregulation in disease has led to ongoing interest in their diagnostic and prognostic potential. Circulating microRNAs may be valuable predictors of early-life complications such as birth asphyxia or neonatal seizures but there are relatively few data on microRNA content in plasma from healthy babies. Here we performed small RNA-sequencing analysis of plasma processed from umbilical cord blood in a set of healthy newborns. MicroRNA levels in umbilical cord plasma of four male and four female healthy babies, from two different centres were profiled. A total of 1,004 individual microRNAs were identified, which ranged from 426 to 659 per sample, of which 269 microRNAs were common to all eight samples. Many of these microRNAs are highly expressed and consistent with previous studies using other high throughput platforms. While overall microRNA expression did not differ between male and female cord blood plasma, we did detect differentially edited microRNAs in female plasma compared to male. Of note, and consistent with other studies of this type, adenylation and uridylation were the two most prominent forms of editing. Six microRNAs, miR-128-3p, miR-29a-3p, miR-9-5p, miR-218-5p, 204-5p and miR-132-3p were consistently both uridylated and adenylated in female cord blood plasma. These results provide a benchmark for microRNA profiling and biomarker discovery using umbilical cord plasma and can be used as comparative data for future biomarker profiles from complicated births or those with early-life developmental disorders.

## Introduction

Complications during childbirth and pre-term births can lead to developmental and neurological dysfunction in later life in a subset of children [1–4]. There remains a major unmet need for molecular biomarkers of maternal and neonatal complications such as hypoxic ischemic encephalopathy (HIE). The development of reliable, non-invasive biomarkers would allow us to identify at an early stage, babies at risk of succumbing to developmental and neurological deficits and enable early intervention or prevention. Umbilical cord blood and plasma are a potential source of pertinent biological information following birth which could contain predictive biomarkers of neurological outcome, however little is known about the molecular profile of umbilical cord plasma.

MicroRNAs (miRNAs) are ubiquitously expressed, short non-coding RNAs which fine-tune gene expression by negatively regulating mRNA translation [5]. They are shuttled between cells via extracellular vesicles [6–8] and importantly are abundant in peripheral biofluids including plasma [9], urine [10], cerebrospinal fluid [11], tears, saliva and peritoneal fluid [12]. They were first profiled in human plasma, serum and microvescicles in 2008 [13–15] and since then, subsequent studies have found that their levels in peripheral biofluids often fluctuate in patients with various types of cancer, neurological disorders, sepsis, liver and cardiovascular disease (reviewed in [16–18]). As such miRNAs have received much interest as potential biomarkers and contain many characteristics which render them ideal biomarker candidates: they are more stable than mRNA as they are resistant to RNase cleavage [19]; expression profiles of miRNAs are often more informative and discriminatory than mRNA profiles; they are abundant and profiling miRNAs is rapid and economical [20]; and their levels often change more rapidly in response to an insult or pathophysiological processes allowing early detection of disease which is critical for progressive illnesses such as cancer, Alzheimer’s disease, epilepsy and early life insults [21–25].

We have previously shown that a number of miRNAs display altered expression in umbilical cord blood from infants following perinatal asphyxia [26]. These miRNAs may, in the future, aid clinicians in providing targeted neuroprotection. We have also shown that miRNA alterations may be used to examine downstream targets and elucidate pathogenesis [27]. Accordingly, identification of miRNAs associated with perinatal and neonatal injury is a priority.

Foetal Growth Restriction (FGR) is a disorder which manifests as a reduction or complete halt of genetically predetermined potential growth of a foetus [28]. The placenta plays a vital role in the correct development and growth of the foetus via provision of essential nutrients and protection from toxins which may affect development and growth and is believed to temporally and abundantly produce miRNAs which are involved in placental development and function [29–31]. The placenta can also produce exosomes in which miRNAs can be found [32]. Placentally-expressed miR-424 may play a crucial role in the development of the placenta and is believed therefore to be associated with FGR. Upregulation was noted in placentae with aberrant vascular development linked to FGR [33]. Downregulation of placental miR-16 and miR-21 has been linked to FGR also. MiR-16 has involvement in both apoptosis and regulation of the cell cycle and may display cell-specific functions and expression. PTEN, the target of miR-21, is normally expressed in the placenta and dysregulation may cause aberrant invasion of the placenta, and reduced migration and growth [30]. Trophoblastic miRNAs regulated by hypoxia are increased in maternal plasma and decreased in placental tissue from FGR cases [32].

Preeclampsia affects up to 8% of all live births worldwide and can result in a high risk of morbidity and mortality for both mother and offspring [29], with approx. 25% resulting in FGR [34]. Inadequate placental oxygenation/angiogenesis may result in consequential hypoxia-ischemia seen in the disorder. Zhu *et al*. [35] reported over 90 differentially expressed miRNAs between preeclamptic and healthy patients. Multiple miRNAs are dysregulated in severe cases of preeclampsia when compared to uncomplicated, healthy pregnancies. These included but are not limited to miR-210, miR-195, miR-181a, miR-411, and miR-377 [29, 34]. Master hypoxamir miR-210 expression levels are raised in both placental tissue and plasma samples from preeclamptic women [36] and can influence multiple pathways in preeclampsia, for example, angiogenesis, mitochondrial dysfunction and immunity [31]. Pineles *et al*. [37] demonstrated that overexpression of miR-210 and miR-182 may differentiate preeclampsia from healthy controls. miRNAs which are responsible for regulation of angiogenic factors such as VEGF are also dysregulated in preeclampsia [34].

Serum miR-323 levels differ from healthy controls in both ectopic pregnancy and spontaneous abortion [38, 39]. Conditions such as gestational diabetes may also be diagnosable with miRNA biomarkers, for example, miR-16, miR-17, miR-19a, miR-19b and miR-20a are dysregulated in the condition when compared to healthy subjects [40].

Many RNA species including miRNAs have been shown to be subjected to editing and modification processes including A-I editing, base modifications, as well as chemical modifications [41–45]. Analysis of deep sequencing data has revealed differences between genomic sequences and RNA sequences, including mRNAs, miRNAs and lncRNAs, the result of RNA editing mechanisms which is a critical function of gene regulation [46, 47]. MiRNAs are targeted by A-to-I editing enzymes (ADARs), where an A base is changed to an apparent G, as well as tailing and trimming modifications which involves the addition or removal of a nucleotide at the 3′ or 5′ end of the miRNA entity which is mediated by TUTases, however other forms of modification are also prominent including 2′O-methylation [47–50]. Modification of miRNAs has been shown to modify miRNA-mRNA targeting, stability and RISC-uptake which can alter gene network dynamics and cellular activity and function. Analysis of RNA editing in plasma is still poorly understood and limited by the lower yields of RNA obtained. However analysis of RNA editing, including miRNA editing in peripheral biofluids may confer additional biomarker potential and sensitivity which would allow greater confidence in biomarker identification as well as provide insight into the function of RNA editing in normal and disease processes.

MiRNA profiling has moved away from high throughput qRT-PCR-based platforms towards the use of RNA-sequencing (RNA-seq), however, few datasets on healthy umbilical cord blood plasma exist for reference purposes. In order to develop reliable biomarkers from umbilical cord plasma, it is important that we gain perspective on the naturally occurring miRNA profiles. Previous studies have used microarrays to profile miRNAs in cord blood from neonates with HIE [26], or used RNA-seq to profile miRNA expression from trios of samples from newborn babies and their parents [51], umbilical cord blood derived cells [52, 53], and cord blood buffy coat layers [54], However, a reference dataset of total miRNA profiles and miRNA editing analysis of healthy umbilical cord plasma has yet to be established.

Here we perform unbiased small RNA-seq on umbilical cord blood plasma from healthy new born infants. We compared the expression of miRNAs between sexes and between the maternity hospitals of origin. We also performed preliminary analyses on RNA editing differences which may exist between sexes in order to obtain a more comprehensive catalogue of miRNA profiles in umbilical cord blood plasma. Interrogation of the miRNA profiles from umbilical plasma revealed a largely stable miRNA profile between male and female, however differences in RNA editing were identified, indicating increased complexity in the miRNA makeup of umbilical cord blood plasma in females compared to males.

## Materials and methods

### Study population

Umbilical cord blood samples were collected from two maternity hospitals: Cork University Maternity Hospital (CUMH) and Karolinska University Hospital (KUH). Consent from parents or guardians of the infants included in the study was obtained according to the Declaration of Helsinki and ethical approval was granted from the Clinical Research Ethics Committee of the Cork Teaching Hospitals, Cork, Ireland and local ethical commitee approval in Karolinska University Hospital. This was a nested study of infants recruited to the BiHiVE 2 study (NCT02019147).

In total, 8 infants were included in this study; 4 males and 4 females (Table 1). All infants were of European descent, singleton, full term, uncomplicated vaginal births. Samples were collected from 4 infants in CUMH (2 males and 2 females) and from 4 infants in KUH (2 males and 2 females). We did not detect any significant difference between male and female infants based on maternal age, parity, gestation, birthweight, head circumference or length. The Apgar score for all infants was above 9 at 1 and 5 minutes.

**Table 1:** Summary of infant demographics and clinical findings, showing the mean and the range for each feature.

Variable	Female (n = 4)	Male (n = 4)	*p*-value
CUMH (n)	2	2	
KUH (n)	2	2	
Maternal age (yrs)	31.75 (25-37)	34.25 (26-41)	0.5733
Parity (n)	1.75 (1-3)	2 (1-3)	0.7049
Gestation (weeks)	41 (39-42)	40 (39-40)	0.2070
Birthweight (g)	3525 (2985-4240)	3693.75 (3375-3980)	0.5994
Head circumference (cm)	35.375 (34-36.2)	35.05 (32-37)	0.7940
Length (cm)	50.75 (48-53)	52 (51-53)	0.4122
Apgar score (1 min)	9 (9)	9 (9-10)	0.3559
Apgar score (5 min)	10 (10)	10 (10)	–

A two-tailed unpaired *t*-test was performed between males and females for each variable and the resulting *p*-value is shown. Gestation is rounded to the nearest week. The median Apgar score is shown rather than the mean.

### Biofluid collection

Umbilical cord blood samples were collected immediately after delivery of all infants in this study and processed within 3 h following strict laboratory SOPs by a dedicated research team who were available 24 h a day. Samples were stored at −80°*C* in a monitored storage facility until analysis. 6 ml of umbilical cord blood was collected into vacutainer tubes from the infants and processed within 3 hours of delivery. The plasma was prepared by centrifuging the tubes at 2400 x g, for 10 minutes, at 4°*C*. The supernatant was collected into an RNAase free tube and extra care was taken not to disturb the buffy coat which contains the white blood cells. Plasma was then collected into 250 *μ*l RNAase free eppendorfs and stored at −80°*C*. The level of haemolysis in the plasma samples was assessed by spectrophotometric analysis using a Nanodrop 2000 spectrophotometer. The absorbance at 414 nm was checked and a cut-off level of 0.25 was used to distinguish haemolysis free samples [55].

### Small RNA library preparation and RNA-seq

200 *μ*l of non-hemolyzed cord blood plasma was used to isolate RNA, using the miRCURY RNA isolation kit (Exiqon) according to the manufacturer’s protocol. RNA was eluted in 25 *μ*l. A standard overnight RNA ethanol precipitation step was then performed and RNA was re-suspended in 10 *μ*l RNase free water to concentrate. RNA integrity and quantity was determined using the small RNA detection kit (DNF-470) on a Fragment Analyzer (Advanced Analytical Technologies). RNA (5 *μ*l) from each sample was then used to construct small RNA libraries using the Illumina TruSeq Small RNA library kit. Due to the small amount of input RNA the protocol was modified slightly (all kit reagents were halved) to prevent extensive adapter dimer formation. Libraries were size selected using a Pippin Prep with 3% agarose dye free cassettes and size selection was validated using a 2100 High sensitivity DNA Bioanalyzer chip (Agilent). The concentration of each library was determined using the HS-dsDNA kit for Qubit, libraries were pooled and pooled libraries were sequenced at the Trinseq Facility at the Institute for Molecular Medicine at St. James Hospital Dublin on an Illumina miSeq.

### Sequencing data processing and differential miRNA analyses

The FastQC [56] program was employed to assess the quality of the reads. The fastq files were then upload to Chimira [44] where count-based miRNA expression data were generated. Sequences were adapter trimmed and mapped against miRBase v21 hairpin sequences [57] allowing up to two mismatches per sequence. Further analyses was performed using R/Bioconductor [58, 59]. The edgeR [60] package was used to identify significantly differentially expressed miRNAs following the protocol described by Law *et al*. [61]. The trimmed mean of M-values (TMM) normalisation method [62] was used for normalisation of the miRNA expression count data. Differential expression analysis was performed using voom [63] and limma [64]. *P*-values were adjusted for multiple testing by controlling the false discovery rate (FDR) according to the method of Benjamini and Hochberg [65]. A miRNA was considered to be differentially expressed if the adjusted *p*-value was ≤ 0.05. Expression data have been submitted to the gene expression Omnibus (GSE119002).

### Modification analysis

Modification analysis of the umbilical cord blood samples was performed using Chimira [44]. Chimira allows for the detection of any non-templated sequences within the input small RNA-Seq samples that are not encoded in the genomic sequence of origin. The output of this pipeline is a comprehensive set of all identified 3′, 5′ and internal modifications (SNPs and ADAR-edits). Each modification is characterised by a non-templated sequence pattern and an index, which determines its position relative to the original sequence. In order to study the differential levels of adenylation, uridylation, guanylation and cytocylation between the female and male samples we have collapsed Chimira’s modification counts into either mono-nucleotide or poly-nucleotide patterns of the same nucleotide (e.g. A, UU, CCC, etc.).

Poly-nucleotide patterns refer to sequences of two or more identical nucleotides and are all grouped together into a single modification type. For example, any ‘CC’, ‘CCC’, and/or ‘CCCC’ modifications are considered collectively as poly-C modifications. A mono-nucleotide modification on the other hand, e.g. ‘C’, stands on its own as a distinct modification pattern. All other isoforms are not included in this analysis and their counts are merged with the counts of the corresponding templated sequences. Finally, we have defined as differentially expressed/modified miRNAs with a fold change in expression or modification level that is > 2 (or < -2) and an associated *p*-value < 0.05. Normalization of miRNA modification counts from male and female samples and identification of differentially modified miRNAs was performed using the DESeq2 software package [66].

## Results and discussion

**Fig 1:**
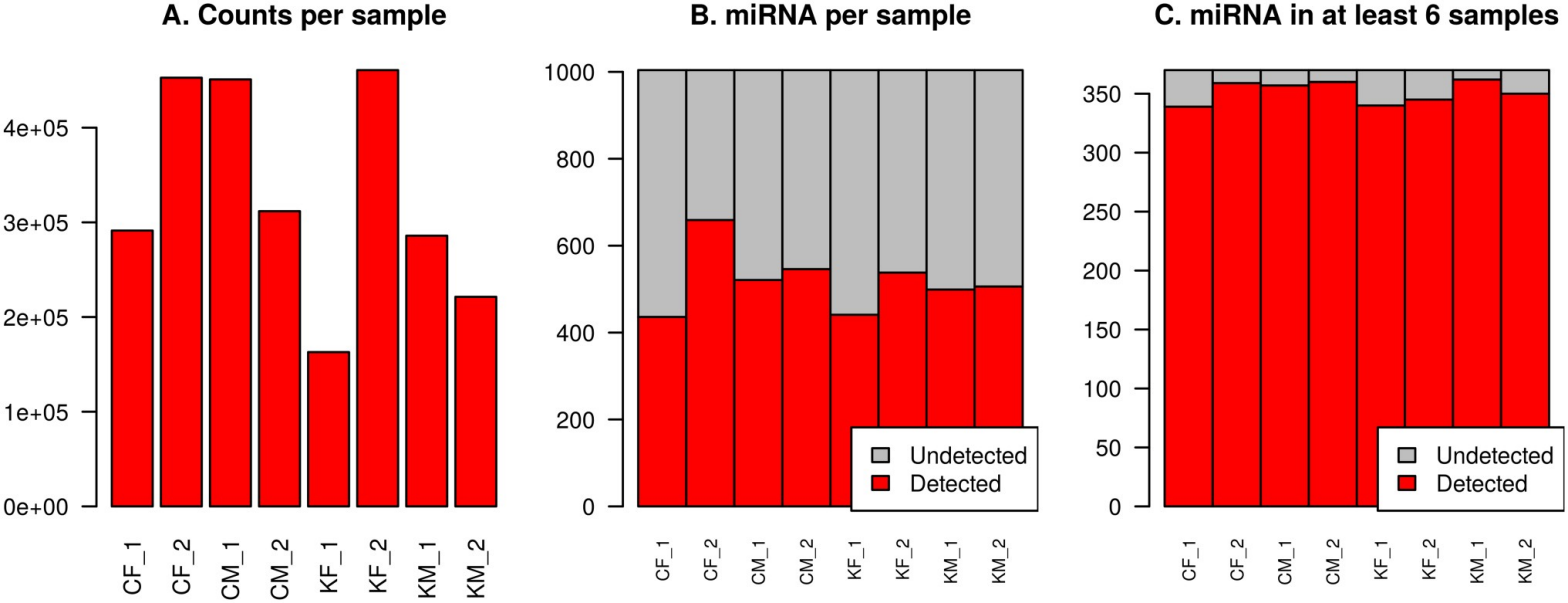
Counts and miRNAs identified per sample. A: Total number of counts per sample mapped to human miRBase V21. B: Number of unique miRNAs with at least one count in any sample. C: Number of unique miRNAs found in at least 6 of the 8 samples. Samples are coded as follows: C—CUMH; K—KUH; F—Female; M—Male.

Count-based miRNA expression data was generated by mapping to human miRBase V21, resulting in an average of 329,721 counts per sample (range 162,954 to 460,783, Fig 1A). A total of 1,004 unique miRNAs were identified across all samples, ranging from 426 miRNAs in sample CF_1 to 659 in sample CF_2 with an average 486 miRNAs per sample (Fig 1B). 370 unique miRNAs were detected in at least six of the eight samples (Fig 1C) and 269 miRNAs were detected in all eight samples (S1 File).

**Fig 2:**
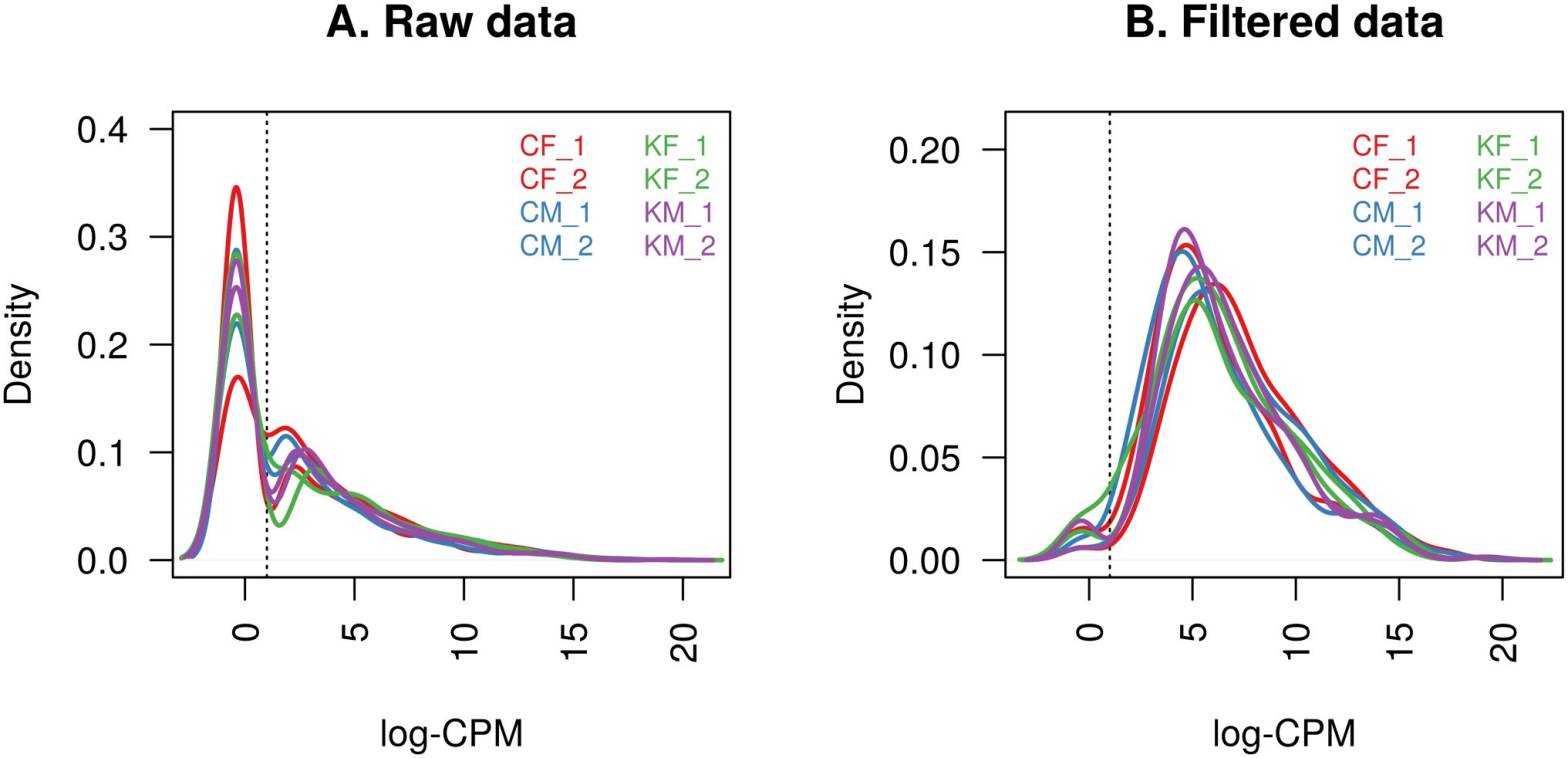
The density of log-CPM values for each sample. A: Raw pre-filtered data B: Post-filtered data The dotted vertical lines mark the log-CPM of one threshold (equivalent to a CPM value of 10) used in the filtering step.

The raw counts were converted to CPM and log-CPM values and miRNAs were removed unless their CPM value was greater than 10 with expression in at least four of the eight samples. Fig 2 shows the density of the log-CPM values for raw pre-filtered data and post-filtered data for each sample. This includes the threshold for the log-CPM of one (equivalent to a CPM value of 10) used in the filtering step. This reduced the number of miRNAs from 1,004 to 300. This is substantially higher than results reported for high throughput qRT-PCR profiling platforms studies of adult plasma [9], and is one of the potential advantages of using an RNA-seq based approach to obtain genome-wide coverage. Lizarraga *et al* [54] used an EdgeSeq miRNA Whole Transcriptome Assay to profile the miRNAs in buffy coat of cord blood samples from 89 newborns, of which 564 miRNAs were retained for further analysis after filtering. Looney *et al* [26] used micorarray profiling of umbilical cord blood of 24 infants retaining 259 miRNAs for differential expression analysis after filtering [26]. Meanwhile, 395 miRNAs were detected in three pooled samples from Down syndrome and normal fetal cord blood mononuclear cells (CBMCs) using RNA-seq expression profiling [52].

**Fig 3:**
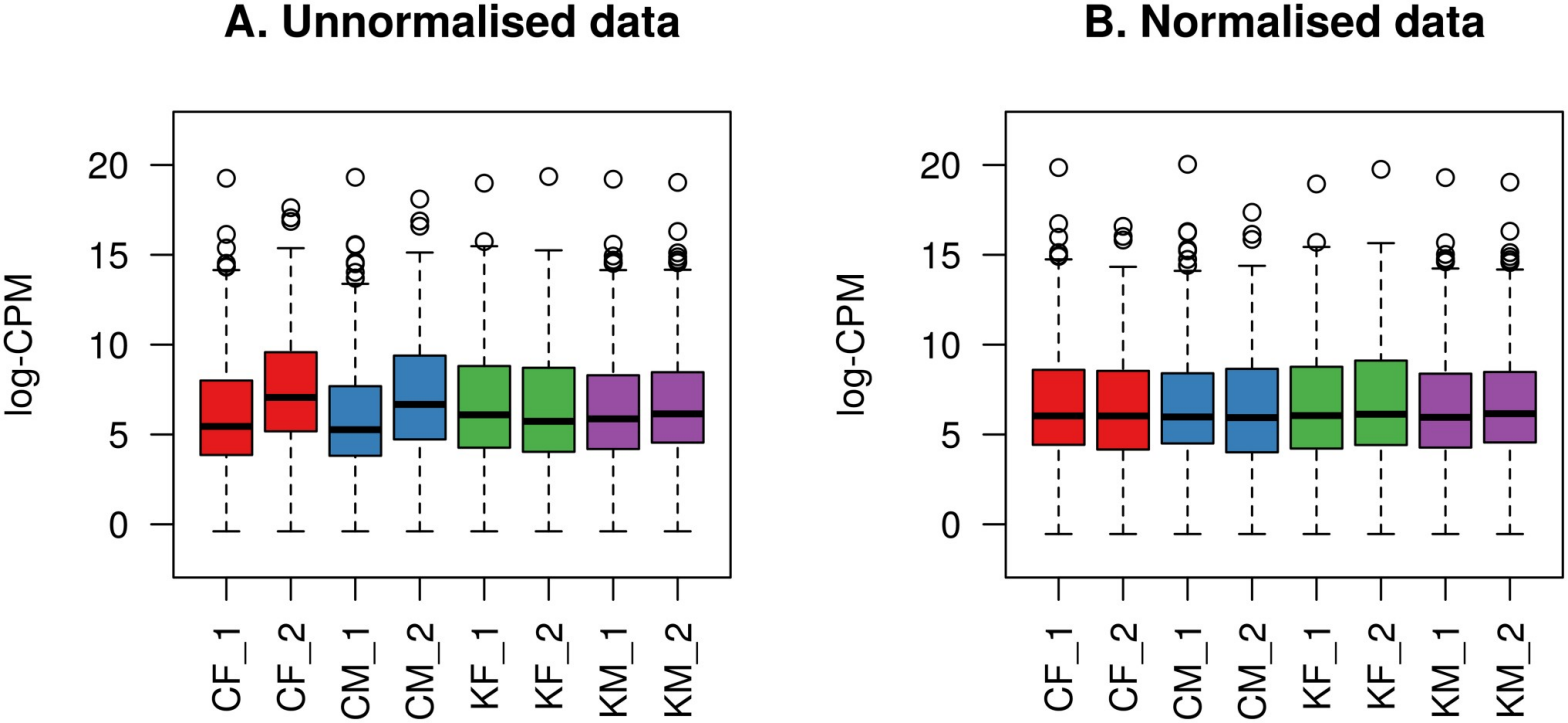
Boxplots of log-CPM values showing expression distributions. A: Filtered unnormalised data for each sample. B: Filtered and TMM normalised data for each sample. The line dividing the box represents the median of the data and top and bottom of the box shows the upper and lower quartiles respectively. The whiskers show the highest and lowest values, excluding outliers, which are show as circles.

The filtered data were then normalised using the TMM method [62]. Boxplots of log-CPM values showing expression distributions for each sample before and after normallisation are shown in Fig 3.

### Unsupervised clustering of samples

Principal Component Analysis (PCA), is a non-parametric method of reducing a complex data set to reveal hidden, simplified dynamics within it. This is accomplished by converting a set of observations of variables (which may be correlated) into a set of values of linearly uncorrelated principal components (PCs). These PCs may then reveal relationships between the variables. PC1 explains the largest proportion of variation in the data, with subsequent PCs having a smaller effect and being orthogonal to the ones before them. Ideally, samples should cluster by the condition of interest, and any outliers should be identified. If the samples cluster by anything other than the condition of interest in any dimensions then that factor can be included in the linear modelling.

**Fig 4:**
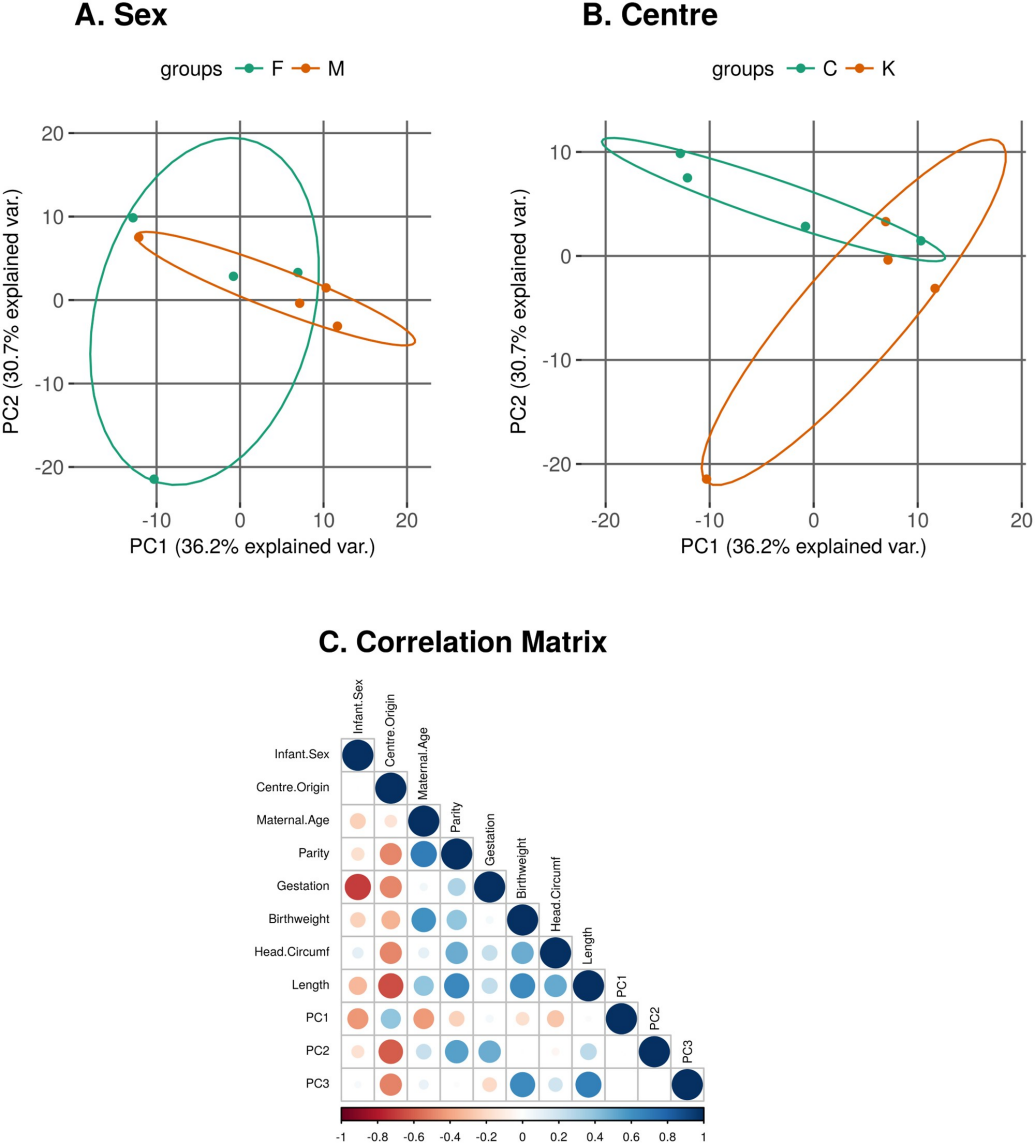
Principal components analysis plots of log-CPM values over PC1 and PC2 showing a 68% confidence ellipse where each point represents a sample. Samples are coloured and labelled by A: Sex of the infant (M/F) and B: Centre of origin of the samples (C—CUMH; K—KUH). C: Correlation Matrix. Positive correlations (Pearson correlation coefficient (*r*)) are displayed in blue and negative correlations in red colour. Colour intensity and the size of the circle are proportional to the correlation coefficients. The PCs > 3 each account for less than 10% of the variation in the data and are not shown.

Fig 4 shows the principal components analysis plots of PC1 against PC2 for the normalised log-CPM values in our data. Each dot represents a sample and we have coloured and labelled the samples by the sex of the infant (Male or Female) (Fig 4A) or the centre of origin of the samples (CUMH or KUH) (Fig 4B). We can see from these plots that there is no obvious clustering by either sex or centre of origin. However, when we calculate the Pearson correlation coefficient (*r*) between the individual PCs and the available clinical/demographic variables (Table 1) we can observe that there is some correlation between the sex of the infant and the centre of origin of the sample and PC1 (*r* = -0.44 and 0.4 respectively) and between the centre of origin of the sample and PC2 and PC3 (*r* = -0.6 and -0.5 respectively) (Fig 4C). Therefore we conclude that there may be a slight “batch” effect due to the centre of origin of the sample and have included this factor in the linear model when looking for differentially expressed miRNAs between males and females. The PCs > 3 each account for less than 10% of the variation in the data and are not shown.

### Differential expression analysis

**Fig 5:**
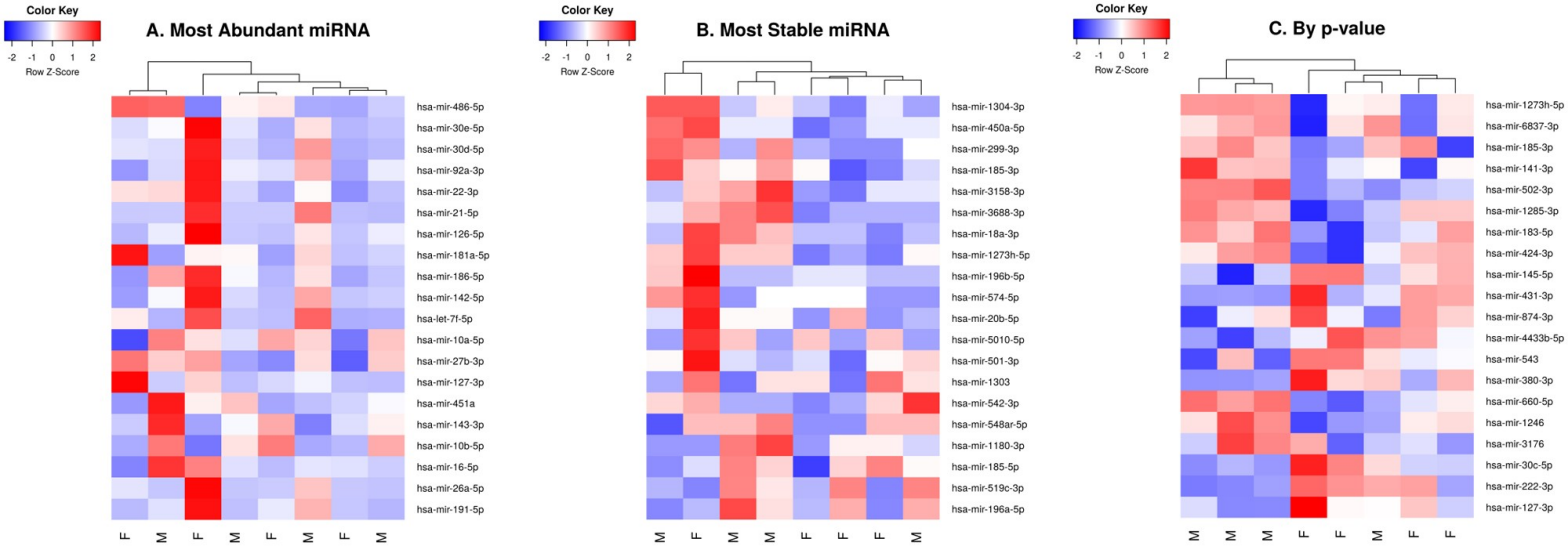
Heatmap of log-CPM values for top 20 miRNAs ranked by: A: Most abundant, B: Most stable and C: By *p*-value following differential expression analysis. Expression across each miRNA has been scaled so that mean expression is zero and standard deviation is one. Samples with relatively high expression of a given miRNA are marked in red and samples with relatively low expression are marked in blue. Lighter shades and white represent genes with intermediate expression levels. Samples and genes have been reordered by the method of hierarchical clustering. A dendrogram is shown for the sample clustering.

Differential expression analysis was performed using voom [63] and limma [64] as implemented in the R package, edgeR [60]. Subsequently, empirical Bayesian moderation was applied by borrowing information across all miRNAs to obtain more precise estimates of miRNA variability. Significance was defined using an adjusted *p*-value [65] cutoff that is set at 5% by default. No miRNAs were found to be significantly differentially expressed between male and female infants (Table 2). A heatmap of log-CPM values for the top 75 miRNAs ranked by *p*-value is shown in Fig 5.

**Table 2:** Differential expression analysis. Males versus females.

	logFC	*p*-value	adj. *p*-value
hsa-miR-145-5p	1.536	0.025	0.894
hsa-miR-141-3p	-1.999	0.031	0.894
hsa-miR-660-5p	-1.699	0.035	0.894
hsa-miR-380-3p	2.805	0.037	0.894
hsa-miR-3176	-1.719	0.045	0.894
hsa-miR-874-3p	1.580	0.046	0.894
hsa-miR-127-3p	1.557	0.048	0.894

Table showing the log fold change (logFC), *p*-value and adjusted *p*-value (adj. *p*-value) of miRNAs with *p*-value < 0.05 (sorted by *p*-value) following linear modelling in limma with empirical Bayes moderation.

### Cellular origin of miRNAs in umbilical cord plasma samples and biomarker potential

The 10 most abundant miRNA (i.e. highest average miRNA expression across all samples) are: miR-486-5p, miR-10b-5p, miR-26a-5p, miR-191-5p, miR-16-5p, miR-22-3p, miR-181a-5p, miR-92a-3p, miR-451a and miR-30e-5p. Many of these are similar to those found in [51]. The most abundant miRNA, miR-486-5p, accounts for nearly 50% of all raw miRNA counts in our samples. miR-486-5p has previously been noted to be highly expressed in RNAseq studies [13, 51] and is detected in most profiling studies of adult plasma that we have seen (S2 File). This miRNA is abundant within red blood cells suggesting that this may be due to selective secretion to plasma or increased stability in plasma [51], however, it does not appear to be detected with such abundance in high-throughput qRT-PCR profiling [9].

Utilising Ensembl [67], the locations of the highest expression of these 300 miRNAs were determined. Of the most abundant miRNAs, interestingly, 49 were most highly expressed in “brain fragments”, 46 in the choroid plexus, 27 in the hindbrain, 21 in the forebrain/forebrain fragment and 11 in the spinal cord. While others were most abundant in the cerebellum (8), the cerebral cortex (8), the medulla oblongata (7), the basal ganglia (6), the temporal lobe (5) or in the midbrain (3). Other miRNAs of note were most abundant elsewhere such as skeletal muscle, ovaries, testis, adrenal gland and stomach. This is interesting as a number of placental miRNAs have been linked to brain development: miR-16-5p, miR-21-5p, miR-93-5p, miR-182-5p, miR-146a-5p and miR-135b-5p [68]. All of these, except miR-135b-5p, were found to be abundantly expressed in our samples (S3 File).

Haider *et al*. [18] have created a miRNA expression matrix spanning 18 cell types, reflecting a broad range of most major cell types (epithelial, endothelial, mesenchymal, hematopoietic, and muscle). We examined the possible cellular origin of the 100 most highly expressed miRNAs in the umbilical cord plasma samples by cross checking them against the 100 most highly expressed miRNAs in each of 18 unique cell types (S3 File). Thirty of the 100 most highly expressed miRNAs were not found in any of the 18 cell types and 19 miRNAs were ubiquitously expressed: miR-26a-5p, miR-16-5p, miR-22-3p, miR-21-5p, let-7f-5p, miR-25-3p, miR-103a-3p, miR-93-5p, miR-26b-5p, let-7a-5p, miR-19b-3p, miR-107, miR-29a-3p, miR-15a-5p, let-7g-5p, miR-27a-3p, miR-23a-3p, let-7d-5p and miR-29c-3p. Thirteen of these ubiquitously expressed miRNAs were also found in our profiling study of adult plasma (let-7d-5p, let-7g-5p, miR-103a-3p, miR-15b-5p, miR-16-5p, miR-19b-3p, miR-21-5p, miR-25-3p, miR-26a-5p, miR-26b-5p, miR-27a-3p, miR-29a-3p and miR-93-5p) [9]. Sixty nine miRNAs were found in at least one and 23 miRNAs were found in all of the seven of the hematopoietic cell types (centroblast, memory B cell, monocyte, naive B cell, NK cell, plasma B cell and red blood cell) (S3 File).

The three most studied pregnancy-associated miRNA-clusters [69] are the chromosome 14 miRNA cluster (C14MC), the chromosome 19 microRNA cluster (C19MC) and miR-371-3 cluster, which is also localized on chromosome 19. The C14MC (also called the miR-379/miR-656 or miR-379/miR-410 cluster) is the largest comprising 52 miRNA genes and is placental mammal lineage specific [69]. C19MC (also known as miR-498(46)) contains 46 miRNA genes. It is primate-specific and is expressed in placenta, embryonic stem cell (ESC), and certain tumors [69–71]. Williams *et al*. found high expression of C19MC in placental tissue and in samples from umbilical cord and mothers but low expression in nonpregnant women and fathers [51]. C19MC is also highly expressed in infantile hemangioma [71]. The third cluster, miR-371-3 cluster, consists mainly of three miRNAs, miR-371a-3p, miR-372 and miR-373-3p, located on chromosome 19 within a region adjacent to the C19MC cluster. Similar to C14MC and C19MC, this cluster is conserved in mammals and is predominantly expressed in the placenta [69]. 10% of the 300 miRNA in our filtered dataset map to these three clusters.

Indeed, many miRNAs identified in our study have been linked with processes associated with pregnancy and pregnancy related complications suggesting that umbilical cord plasma miRNA profiles may reflect pregnancy status and potential complications. For example, miRNA associated with preeclampsia (let-7a-3p, miR-24-3p, miR-26a-5p, miR-29a-3p, miR-103a-3p, miR-125a-5p, miR-125b-5p, miR-130b-3p, miR-181a-5p, miR-342-3p miR-542-3p and miR-574-5p) [72–74] and ectopic pregnancy (miR-323a-3p) [39].

### Comparison of umbilical cord plasma miRNA profiles to adult miRNA plasma profiles

In a previous study of miRNA expression profiles in adult plasma we found that although there is a similar number of miRNAs being detected across studies, there is a large degree of variation between the lists of miRNAs being detected by the different platforms (e.g. high throughput qRT-PCR profiling or RNA-seq, Exiqon or TaqMan, etc) [9]. However, we observed that there was a set of 40 miRNAs that were common to at least six of the seven studies that were compared [9, 12, 13, 15, 75, 76]. In this case, of the 300 miRNAs that remained in our study after filtering, 192 of these have been previously detected in at least one other profiling study of adult plasma (S2 File).

Of the 108 miRNAs which were not detected in adult plasma 11 of these miRNA are also in the top 100 most abundant miRNA in our samples: miR-381-3p, miR-378a-3p, miR-92b-3p, miR-654-3p, miR-106b-3p, miR-6131, miR-340-5p, miR-151b, miR-1307-5p, miR-421 and miR-3182. This could indicate that umbilical cord plasma has a unique miRNA profile which may contain biomarkers more selective for pre/post-natal development or disease. This may also reflect the temporal changes in miRNA expression throughout ageing and future studies may compare miRNA profiles from adolescents and elderly people to determine this further.

The NCBI GeneRIF database [77] was used to determine whether any of the 108 miRNAs that were not found in adult plasma played specific roles in pregnancy or pregnancy complications. We identified miRNA that are involved throughout pregnancy from the preparation of the endometrium for pregnancy (miR-181a-3p) [78], to embryo attachment and early development (miR-145) [79, 80], embryonic stem cell differentiation and renewal (miR-181a-2-3p and the let-7 family of miRNAs) [81, 82], umbilical cord derived mesenchymal stem cells proliferation (miR-26b-3p) [83] and placental growth (miR-377-3p) [84]. Additionally, miRNAs including miR-141, miR-145, miR-378a-3p and miR-424 were identified that are regulators of trophoblast invasion, proliferation, survival and differentiation [79, 84–87].

Alterations in many of the miRNA that are present in our samples that were not detected in adult plasma have been linked to complications in both mothers and foetuses. These miRNA include: miR-18a, miR-136, miR-221, miR-141 and miR-145, which have been implicated in preeclampsia [88–91]. Additionally, miR-141 has been implicated in unexplained recurrent spontaneous abortions [92], miR-424 and miR-141 have been linked with fetal growth restriction [33, 93]) and miR-374a-3p is downregulated in HIE [26].

Using miRTarBase [94], we identified 75 validated targets (supported by strong experimental evidence) of the most abundant miRNA in our samples that were not found in adult plasma (summarised in Table 3)). We found evidence to support the role of 50 of these genes in pregnancy and pregnancy related complications.

**Table 3:** Validated gene targets of most abundant miRNA.

hsa-miR-378a-3p	hsa-miR-340-5p	hsa-miR-92b-3p	hsa-miR-381-3p
CDK6 [95, 96]	AKT1 [97, 98]	CDKN1C [99–101]	ANO1
CYP19A1 [102]	CCND1	DAB2IP [103, 104]	CD1C [105]
GALNT7	CCND2y [96]	DKK3 [106, 107]	GJA1 [108, 109]
GLI3 [110]	CCNG2 [111, 112]	ITGA6 [113, 114]	HDAC4
GOLT1A	CDK6 [95]	ITGAV [115]	ID1
GRB2 [116, 117]	HNRNPA2B1 [118]	NLK	NFKBIA
IGF1R [119]	IL4 [120]	PRMT5 [121]	P2RX5
KSR1	KRAS [122]	PTEN [123, 124]	TBC1D9
MAPK1 [125, 126]	MDM2 [127, 128]	RAB23 [113, 129]	TWIST1 [130, 131]
MSC	MECP2 [132]	RECK [133, 134]	WEE1
MYC [135]	MET	SLC15A1	
NPNT	MITF [130]	SMAD3 [136]	
PGR [137]	PTBP1	SMAD7 [88]	
RUNX1 [138]	PUM1 [139, 140]		
SUFU	PUM2		
TGFB2 [141]	RHOA [142, 143]		
TOB2 [144]	ROCK1 [115]		
TUSC2 [145]	SKP2 [146]		
VEGFA [147]	SOX2		
VIM	STAT3 [148–150]		
WNT10A			
hsa-miR-421	hsa-miR-106b-3p	hsa-miR-654-3p	
ATM	BMP2 [123]	CDKN1A [151–153]	
CASP3 [154]	PTEN [123, 124]		
CBX7			
CDH1 [155]			
FOXO4 [156–158]			
RBMXL1			
SIRT3 [159]			
SMAD4 [160]			

Table showing the 75 validated targets, found using miRTarBase [94], for the most abundant miRNA in our samples that were not found in adult plasma. Where available, the citation which supports a link between the gene and pregnancy is show.

Many of the genes that we identified as targets of these miRNA are believed to play a role in the preparation and development of the uterus in early pregnancy (TWIST1 [130], CDK6 [95], RUNX1 [138], CDK6 [95], MITF [130], HNRNPA2B1 [118] and BMP2 [123]), decidualisation (CDKN1A [153] and STAT3 [150]), implantation of the embryo (RECK [134], KRAS [122], CDH1 [155] and SMAD4 [160]) and the activation of the migration, invasion, proliferation and differentiation of the trophoblast (MAPK1 [125], MYC [135], TGFB [141], DAB2IP [103], RECK [133], SMAD7 [88] and STAT3 [149]). They continue to play roles during pregnancy and are important for the healthy progression of pregnancy and fetal growth (CASP3 [154], GLI3 [110], VEGFA [147], CCND2 [96], CDKN1C [99], IL-4 [120], IGF1R [119] and Akt1 [98]). DKK3 [107], ITGAV [115], TWIST1 [131] and ROCK1 [115] upregulation has also been reported in the myometrium in healthy pregnancies at full-term. While CCNG2 [96] and FOXO4 [156] are downregulated in the placenta at full term. A number of these genes have been implicated in complications of preganacy including: miscarriage (VEGFA [147], DAB21P [103], CDKN1A [151, 151, 152], PTEN [124] and MDM2 [128]); preeclampsia (CD1C [105], CYP19A1 [102], VEGFA [147], CDKN1C [100, 101], DAB2IP [104], CCNG2 [111] and GRB2 [116]); pre-term labour (RhoA [142, 143]); gestational diabetes (CCNG2 [111, 112], ITGA6 [113], RAB23 [113] and FOXO4 [158]); gestational trophoblastic disease (MDM2 [127]); male-specific neonatal encephalopathy (MECP2 [132]), neural tube defects (ITGA6 [113] and RAB23 [113]); Carpenter Syndrome (RAB23 [129]); intrahepatic cholestasis of pregnancy (PUM1 [139]); ectopic pregnancies (DKK3 [106]); and foetal conotruncal anomalies (MAPK1 [126].

### Analysis of miRNA editing

**Fig 6:**
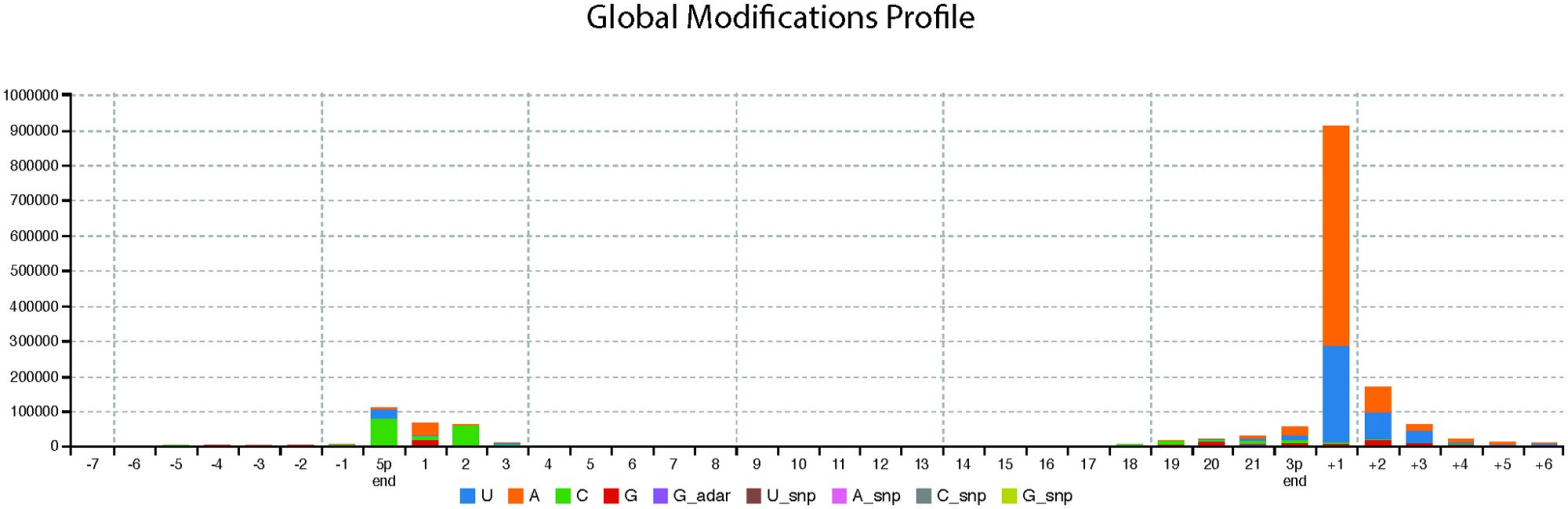
Global modification profile of all samples showing high levels or adenylation and uridylation at the 3′ end of the miRNA. The x-axis corresponds to the index positions across a miRNA molecule. They y-axis corresponds to the raw counts of the identified modification patterns. The start of a miRNA on the x-axis is at index ‘0’ (5′ end) while its end is at index ‘22’ (3′ end).

**Fig 7:**
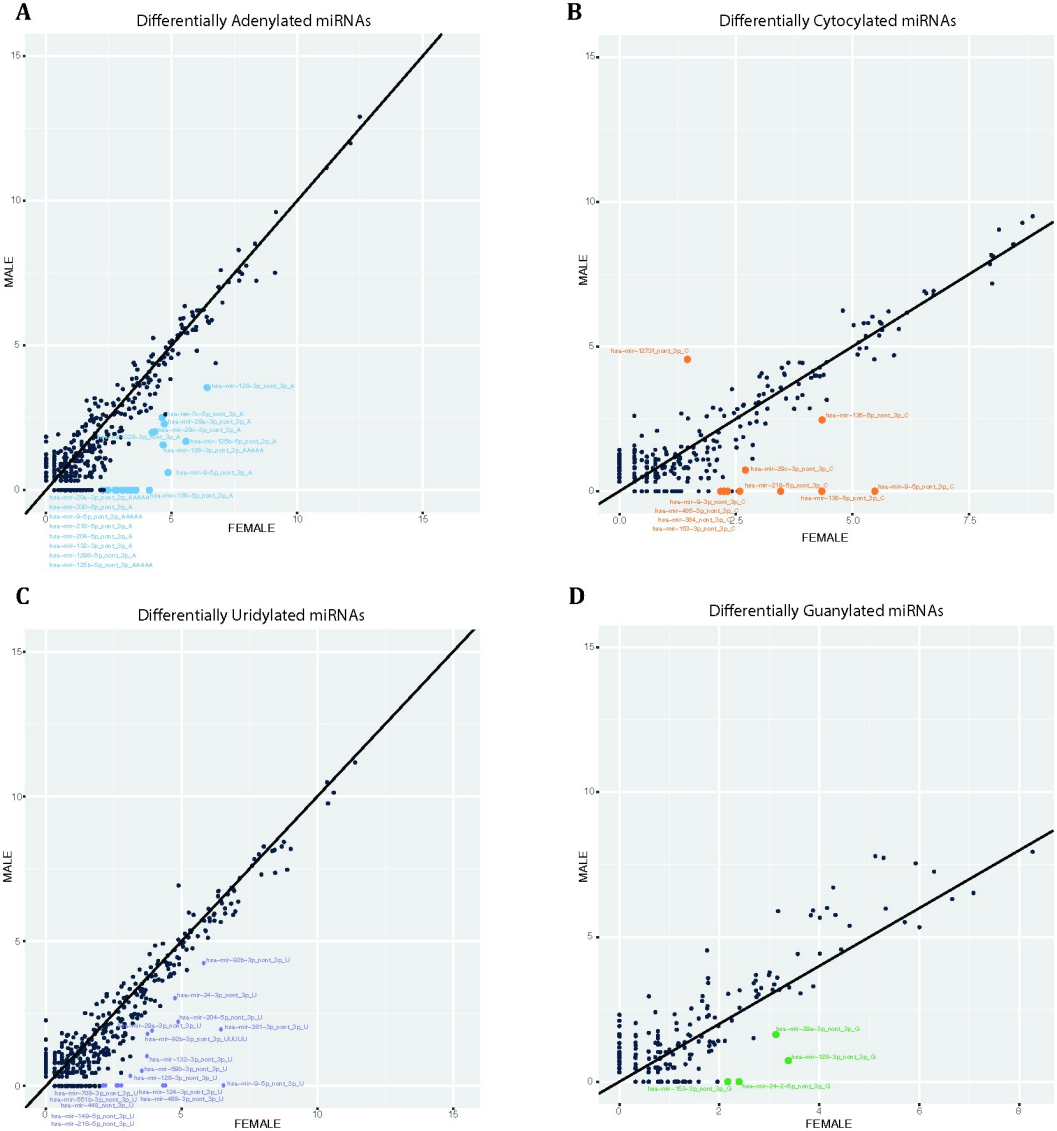
Differential expression of modified miRNAs between female and male samples (log_2_ of count data). Statistically significant differentially expressed miRNAs are highlighted in non-black. A: Adenyated, B: Cytocylated, C: Uridylated and D: Guanylated.

RNA editing enzymes including ADAR proteins have been shown to function aberrantly in various types of cancers and neurological disorders [161–164]. Additionally, it has recently been demonstrated that it is possible to distinguish between different cancer types based on the presence or absence of alternatively modified miRNAs (isomiRs) [165]. As such it is intuitive that the search for biomarkers should include criteria which would allow the identification of alternatively edited RNAs, as they may correlate with, and therefore aid in diagnosis of disease development, progression and prognosis [165]. MiRNAs have been shown to be subjected to edits and modifications and we therefore expanded our interrogation of umbilical cord plasma miRNA profiles to investigate the prevalence of editing and whether differences exist between sexes. Using Chimira [44], we performed editing analysis on our sequencing data, initially analysed global modification profile of all samples and differential expression of adenylated, uridylated, guanylated and cytocylated miRNAs (Fig 6). Statistically significant differential expression was taken as a fold change in expression of > 2 (or < -2) and an associated *p*-value < 0.05. We found there were consistently more differentially expressed edited miRNAs in female cord blood compared to males (Fig 7). However, expression of the differentially edited miRNAs is very low and thus further evidence is required in order to infer any functional implications from the differential modification profiles in this particular study (S4 File). Of the four types of editing analysed adenylation and uridylation were the most abundant differential modification identified, which is in line with previous editing analysis [45, 166–169]. Of note, miR-128-3p, miR-29a-3p, miR-9-5p, miR-218-5p, 204-5p and miR-132-3p were consistently both uridylated and adenylated in female cord blood plasma (S1 Fig).

Of further interest was the origin of miRNAs which were both differentially adenylated and uridylated in females. With the exception of miR-29a-3p (which is enriched in brain) 5 of the 6 miRNAs which underwent differential adenylation and uridylation are brain specific [170]. Editing of RNA has been reported to be highly prominent in the central nervous system [171]. It is interesting to speculate on the origin of these miRNAs, as these are healthy infants who experienced routine vaginal births, leakage via a disrupted blood brain barrier is unlikely. It is possible however that these brain specific miRNAs were encapsulated in signalling micro-vesicles and transported out of the brain in order to elicit a peripheral cell response [172]. Site-of-editing analysis revealed that editing occurred most frequently at the 3′ end of the miRNA molecule. Specifically adenylation and uridylation occurred most frequently at the +1 site suggesting 3′ tailing of the miRNAs. MiRNAs have been shown to be selectively uridylated by 3′ terminal uridylyl transferases (TUTases) TUT7 and TUT4. This editing can modify miRNA-gene regulatory networks by affecting the stability of the miRNA [50, 173–175].

## Conclusion

miRNAs play a role in multiple key processes throughout pregnancy; including preparation of endometrial tissue for implantation, management of immune-associated genes, development of the placenta and angiogenesis [28]. Dysregulation of the expression of these miRNAs may therefore be associated with complications in pregnancy [29], making them good candidate biomarkers for not only HIE [26, 176], but for many pregnancy-related disorders. Furthermore, due to the relative stability of miRNAs under normal conditions [177], they appear to be potentially useful diagnostic biomarkers of multiple disordered states [178] in pregnancy and beyond. More research is required however, to decipher their target pathways and mechanisms of action.

While overall miRNA expression did not differ between male and female cord blood plasma, we did detect differentially edited miRNAs in female plasma compared to male. Editing of miRNAs is now known to be altered in disease [161–164] and can affect miRNA-mRNA targeting, indeed Choudhury *et al*, identified that A-I editing of miR-376 was reduced in glioma and had an effect on the repertoire of target mRNAs [179]. This allowed an increase in cell invasiveness. As such it is intuitive that future miRNA biomarker studies profile changes in miRNA editing as it may correlate with disease development, progression and outcome. Of note, and consistent with other studies of this type, adenylation and uridylation were the two most prominent forms of editing. Analysis of the sites of adenylation and uridylation along the miRNA molecule revealed that editing was most prominent at the 3′ end of the miRNAs at the +1 position, indicating 3′ tailing, a common modification of miRNAs. Analysis of the expression patterns of these miRNAs revealed that all except miR-29a-3p are expressed almost exclusively in brain [170]. Although only a few miRNAs were differentially edited in females and expression levels were low, it is an interesting finding as the effects of sex on RNA editing is poorly understood and warrants further investigation.

This study is the first to profile miRNA editing in cord blood plasma from healthy infants. Although we did not detect a difference between male and female miRNA expression, possibly due to the small sample size, and expression of the differentially edited miRNAs is very low, this study can be used as comparative data for future biomarker profiles from complicated births or those with developmental disorders including those initiated by HIE.

## Supporting information

S1 Fig Expression patterns of the miRNAs which were both differentially adenylated and uridylated in female cord blood plasma. A: miR-128-3p, B: miR-29a-3p, C: miR-9-5p, D: miR-218-5p, E: 204-5p and F: miR-132-3p. Analysis of the expression patterns of these miRNAs revealed that all except miR-29a-3p (although it is enriched) are expressed almost exclusively in brain (highlighted with green boxes) [170].
(PNG)

S1 File Table showing the raw counts for the 269 miRNA identified in all umbilical cord plasma samples. miRNA are ranked by average abundance across all samples.
(CSV)

S2 File miRNA identified in the umbilical cord plasma samples after filtering and their overlap with miRNA identified in six other studies of biofluids in healthy adults. miRNA names were mapped to the mature human sequences from miRBase version 21 [57]. Blondal [75], Chen [13], Mitchell [15], Mooney [9], Wang–Exiqon [76], Wang–Taqman [76] and Weber [12].
(CSV)

S3 File Possible cellular origin of the 100 most abundant miRNAs identified in the umbilical cord plasma samples. a—acinar cell; b—adipocyte; c—ductal cell; d—endothelial; e—epithelial cell; f—fibroblast; g—hepatocyte; h—lymphatic EC; i—myocyte; j—neutrophil; k—smooth muscle cell; l—centroblast; m—memory B cell; n—monocyte; o—naïve B cell; p—NK cell; q—plasma B cell; and r—red blood cell. Cell types a—k are hematopoietic; Cell types l—r are hematopoietic. An asterix is plased in the column if the miRNA is found in the top 100 of miRNAs expressed in that cell type. Expression profiles for all cells taken from [18]. MiRNAs are included if they are identified in at least one cell type and are listed in order of average abundance across all samples (mean). SD—standard deviation.
(CSV)

S4 File Table showing the modification counts for all miRNA identified in any umbilical cord plasma samples. (CSV)
