# Supplementary figures and images for: RNA-sequencing analysis of umbilical cord plasma microRNAs from healthy newborns
Source: PLoS One. 2018 Dec 3;13(12):e0207952. doi: 10.1371/journal.pone.0207952 (PMC6277075; doi:10.1371/journal.pone.0207952)

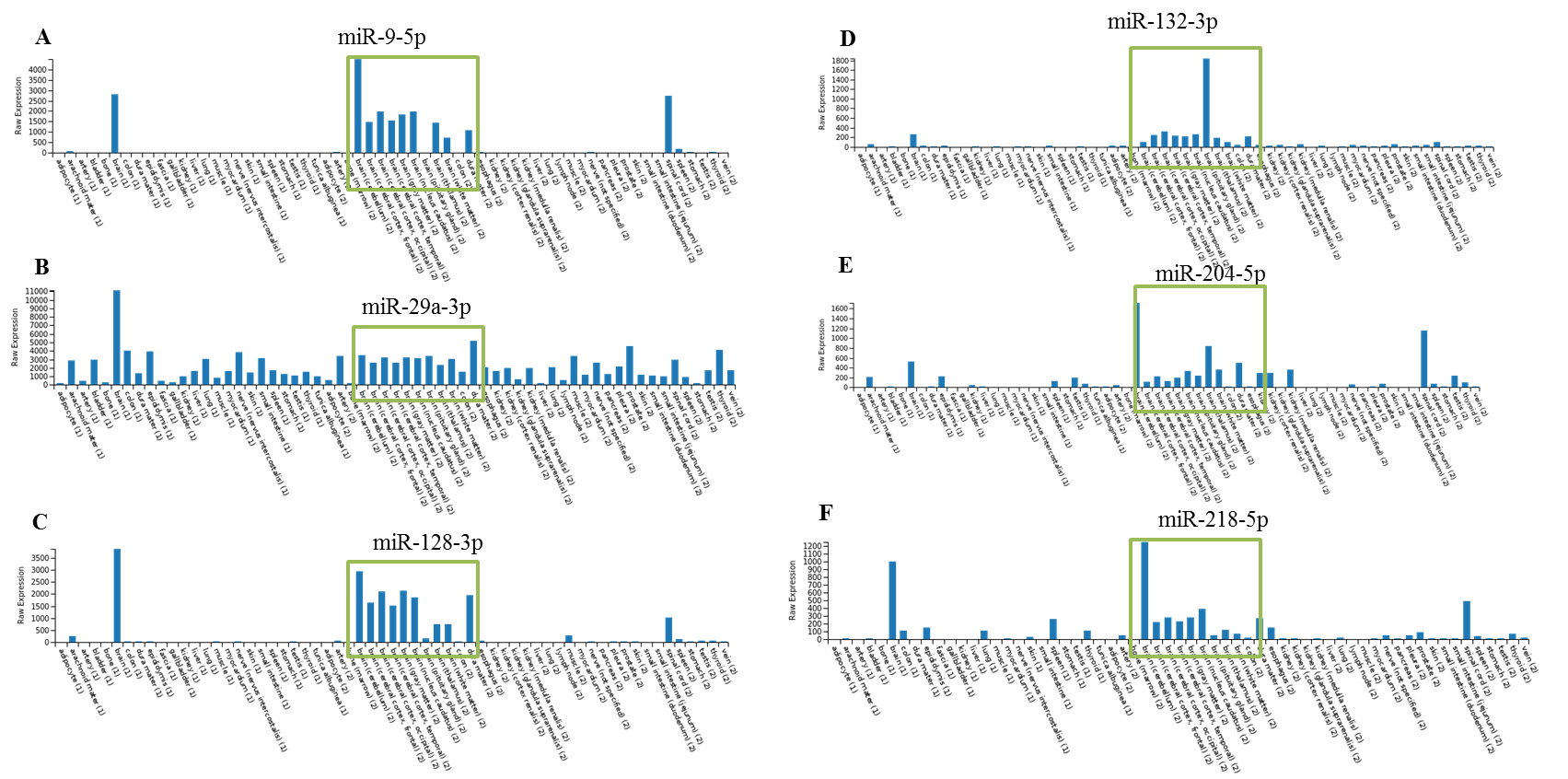

Supplement: S1 Fig — A: miR-128-3p, B: miR-29a-3p, C: miR-9-5p, D: miR-218-5p, E: 204-5p and F: miR-132-3p. Analysis of the expression patterns of these miRNAs revealed that all except miR-29a-3p (although it is enriched) are expressed almost exclusively in brain (highlighted with green boxes) [170]. (PNG) [file pone.0207952.s001.png]
